# A group-based approach to stabilisation and symptom management in a phased treatment model for refugees and asylum seekers

**DOI:** 10.3402/ejpt.v4i0.21407

**Published:** 2013-12-20

**Authors:** Mary E. A. Robertson, Jocelyn M. Blumberg, Jacqui L. Gratton, Eileen G. Walsh, Hamodi Kayal

**Affiliations:** Traumatic Stress Clinic, Camden and Islington NHS Foundation Trust, London, UK

**Keywords:** complex PTSD, refugees, phased treatment model, stabilisation

## Abstract

**Background:**

Traumatised asylum seekers and refugees may present with significant and complex mental health problems as a result of prolonged, extreme, and multiple traumatic events. This is further complicated by ongoing complex social circumstances.

**Concepts:**

In our work at the Traumatic Stress Clinic (TSC), the understanding afforded by the concept of complex posttraumatic stress disorder (PTSD) together with the related notion of a phased treatment model, provides a useful framework for organising our work with this population.

**Clinical Applications:**

An explication of complex PTSD as it applies to our client group is presented, followed by a description of our phased treatment model and an outline of the core principles, which guide our clinical approach. Our symptom management and stabilisation groups have been developed and refined over time and draw on techniques from a variety of cognitive behavioural therapies. These are described in some detail with illustrative clinical case vignettes.

**Conclusion:**

This paper concludes with some reflections on the challenges inherent to working with this complex client group.

The Traumatic Stress Clinic (TSC), Camden & Islington Foundation NHS^1^ Trust, is a specialist service for the psychological treatment of posttraumatic stress disorder (PTSD) for adults living within the London Boroughs of Camden and Islington. Service users come from diverse ethnic and social backgrounds and include a large refugee population.

Approximately, 51% of clients seen within the TSC are refugees and asylum seekers (RAS) from a range of countries. Clients have generally experienced multiple traumatic events across the lifespan including but not limited to:

- Women trafficked and held captive for the purposes of domestic servitude and/or sex
- Survivors of prolonged imprisonment and torture
- Survivors of prolonged political violence and war, with attendant multiple losses
- Survivors of multiple rape

These clients often present with co-morbid difficulties including depression, chronic pain, substance misuse, dissociative disorders, psychosis and mild brain injuries.

In this article, we explicate the concepts of complex PTSD and a phased treatment model, which we find useful in understanding and working with RAS clients. Given our clients’ particular experiences, we highlight certain central principles which inform our approach. This article focuses primarily on our phase one group treatment, aimed at symptom management and stabilisation.

## Complex PTSD

There is growing recognition that existing PTSD diagnostic criteria fail to encapsulate the more chronic, complex, and far-reaching symptoms and experiences of survivors of prolonged and repeated interpersonal trauma (e.g., Herman, 1992; van der Kolk, Roth, Pelcovitz, Sunday, & Spinazzola, 2005). According to Herman (1992), complex PTSD generally results from prolonged interpersonal and/or social traumatic events often occurring under circumstances where escape is impossible due to physical, psychological, emotional, maturational, environmental, and social constraints. This includes prolonged childhood abuse, domestic violence, and situations of repeated trauma in captivity, such as that endured by political prisoners who are repeatedly tortured and women trapped in trafficking situations.

Herman (1992) most notably argued for a new diagnosis of complex PTSD, to more accurately describe the presentations and experiences of such individuals, facilitating more effective clinical treatment. More recently, PTSD researchers and clinicians have set about explicating the core features of Complex PTSD with a view to publishing more clinically relevant and useful diagnostic criteria.

For example, the International Society for Traumatic Stress Studies’ Complex Trauma Task Force (CTTF) agreed on a new working definition of complex PTSD. The new definition and recently developed clinical guidelines for working with complex PTSD (Cloitre et al., 2012) resonates with the clinical presentations of our RAS clients and our approach to working with this client group.

The CTTF definition includes the three core symptom clusters of PTSD (re-experiencing, avoidance/numbing, and hyper-arousal) together with a range of disturbances in self-regulatory capacities across five domains (Cloitre et al., 2012):

### Difficulties with emotion regulation

Many of our clients present with histories of aggressive outbursts and self-destructive behaviours as well as extreme anxiety and agitation. This is common in torture survivors who frequently report chronic emotional dysregulation.

### Disturbances in relational capacities

RAS clients with complex PTSD may present with marked and entrenched feelings of mistrust, a profound sense of isolation and feeling cut-off from others. This can present barriers to trusting interpreters, establishing a therapeutic relationship, tolerating regular sessions or managing a group setting.

In addition, mistrust of others within the community may prevent RAS clients from connecting with individuals/services that could potentially support integration, for example, accessing English classes.

### Alterations in attention and consciousness (such as experiencing dissociation or depersonalisation)

RAS sufferers of complex PTSD often present with severe dissociative episodes in which they vividly re-experience the traumatic event, losing contact with the present completely (dissociative flashbacks). These episodes are usually triggered under stress and can be prolonged. During therapy, this can manifest as the client appearing to lose touch with time, place and context and being inaccessible to regular grounding strategies. Such clients frequently put themselves and others at risk due to being unaware of their own actions. In such cases, the clinician has to work with the client to identify possible triggers to dissociation so as to support them in keeping safe, as well as enabling them to remain grounded during treatment.

Other unusual flashbacks that we see in our RAS clients include:

- Auditory flashbacks: whereby a client reports hearing sounds or voices, which are not there. This may include repeatedly “hearing” the screams of others being tortured, cell doors slamming, or the abusive remarks of torturers. Without an understanding of the clients’ trauma history it can be difficult to make links to actual experienced events and clients can be misdiagnosed as psychotic.
- Emotional flashbacks: involve sudden and often prolonged, de-contextualised regressions to the frightening feeling states experienced during past traumatic events.
- Somatic flashbacks: bodily memories in which the individual re-experiences the traumatic event in a physical manner. For example, feelings of being suffocated, beaten or, burning sensations.

### Somatic distress and disorganisation

RAS clients with complex PTSD regularly present with apparent chronic physiological dysregulation such as severe disruptions in sleep with long periods of sleeplessness or going for days without eating. In addition, chronic body pain, not easily attributable to, or proportionate with the effects of previous injuries, is common. Frequent headaches and gastro-intestinal problems are often noted, as is general health anxiety and the subjective, felt sense of being permanently internally damaged, often observed in rape survivors. Chronic high arousal levels contribute to these presentations. Other unexplained medical symptoms in this population are also common, including seizure-like episodes or apparent narcoleptic attacks, with no identified organic cause and which we tend to understand as dissociative phenomena.

### Changes in belief systems

RAS clients with complex PTSD are frequently burdened by very negative self-concepts characterised by intense guilt, shame, and overwhelming feelings of defeat and worthlessness. Similarly, some clients can present with fundamentally changed world views, for example, describing themselves as having formerly been religious but now having lost their faith or as formerly tolerant but now being racist.

#### Treatment implications

This symptom profile highlights the loss of emotional, social, cognitive, and psychological competencies that failed to develop properly due to chronic early trauma and disrupted attachments or that subsequently deteriorated due to prolonged exposure to trauma. The treatment for complex PTSD therefore necessarily emphasises not only the reduction in the core PTSD symptoms, but also equally the development, or restoration and strengthening of the survivor's capacity for self-regulation, self-efficacy and interpersonal, social and environmental functioning (Allen, 2001; Cloitre et al., 2012). It has been noted that clients who have difficulties with emotional regulation, interpersonal relationships and those who are prone to dissociate under stress do not respond as well to exposure-based treatments (Cloitre, Koenen, Cohen, & Han, 2002; Herman, 1992).

In recognition of this, a survey of expert clinicians endorsed a phase-based approach to the treatment of this client group (Cloitre, Courtois, Carapezza, Stolbach, & Green, 2011). This recognises that it is not possible to resolve trauma when survivors are still living in unsafe or traumatising environments and/or when as a result of their experiences of interpersonal cruelty, they lack the capacity to trust others, making engagement in an effective therapeutic relationship difficult.

## Phased model of treatment

A phased model of treatment has also been recommended for treating RAS (Gorman, 2001; Grey & Young, 2007; NICE guidelines, 2005; Nickerson, Bryant, Silove, & Steel, 2011). This approach recognises that these clients have often experienced repeated and prolonged traumatic events and may still be facing situations of ongoing threat and uncertainty related to unresolved asylum claims, missing family members, and other unmet psychosocial needs.

Our phased model is an adaptation of the work of Judith Herman (1992). The three phases of treatment include symptom management and stabilisation (phase one), trauma-focused therapy (phase two), and reintegration (phase three). There is frequent alternation between the phases according to need. 
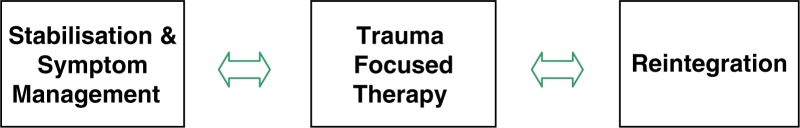


Research evaluating a phased model of treatment with RAS clients presenting with complex PTSD is extremely limited. There are some studies investigating phase-based or enhanced trauma treatment models with other client groups such as those who have complex PTSD as a result of child abuse (Cloitre et al., 2012). Cloitre et al. (2010) directly compared a phase-based treatment (skills training followed by memory processing) to an exposure-focused treatment and to a skills-focused treatment. Results of this study showed that a combined skills training and trauma-focused treatment approach yielded the best results.

Whilst we need to be cautious when applying evidence from a Western client group to the RAS population, this study does offer support for a phased treatment model in the absence of more relevant studies.

### Phase one

This phase of treatment focuses on stabilisation, symptom management, and psycho-education in order to build a sense of safety and trust. A detailed description of our group-based approach to this treatment is the focus of this article.

#### Stabilisation

Stabilisation involves ensuring that the person has sufficient security in their daily life to enable them to engage with and tolerate trauma-focused psychological treatment. This is not possible when basic needs are unmet. In these cases, the following is appropriate: referral for help with legal, financial, and housing problems; with family tracing and reunification issues; or for assistance with other health problems.

#### Psycho-education

An important function of psycho-education is to normalise symptoms and problems that a person may find confusing and frightening and therefore a source of shame or self-criticism. The collaborative identification and formulation of events and situations that trigger symptoms such as flashbacks and intrusive memories, helps to make these phenomena less distressing. As such, this work can facilitate a greater sense of safety and stability.

#### Symptom management

Symptom management aims to promote a sense of self-efficacy, mastery, and control over distressing and incapacitating symptoms. It could include identifying relaxation or self-soothing skills; grounding techniques to manage dissociation, flashbacks and nightmares; coping skills for difficult feelings, such as anger, guilt and shame, and techniques for the management of hyper-arousal.

### Phase two

The second phase of treatment involves the processing of traumatic memories. During this phase, traumatic memories are reviewed so that they are integrated into adaptive representations of self, relationships, and the world (Cloitre et al., 2012). Exposure-based treatments are typically recommended at this stage (Bisson et al., 2007; Crumlish & O'Rourke, 2010; Kruse, Joksimovic, Cavka, Woller, & Schmitz, 2009). We use a combination of trauma-focused cognitive behavioural treatment (Ehlers & Clark, 2000; Grey & Young, 2007), eye movement desensitisation and reprocessing therapy (EMDR) (Shapiro, 1995) and narrative exposure therapy (Schauer, Neuner, & Elbert, 2005). At this stage, frequent reinforcement of phase one work is required, in order to keep clients sufficiently grounded and to keep arousal at optimal levels for effective trauma processing.

### Phase three

A sense of belonging within a community coupled with access to education and employment are all associated with better mental health outcomes for RAS clients (Carswell, Blackburn, and Barker, 2011). Phase three therefore focuses on helping clients integrate into their community (NICE, 2005) so that they are able to resume everyday activities, relationships, work and family life, with the traumatic memories integrated into their life story. This involves focusing on the present and future and may include facilitating access to language skills, education, and employment. Interventions aimed at facilitating integration are usually delivered in partnership with other relevant community-based organisations and services.

Aspects of integration are frequently addressed throughout treatment. However, PTSD symptoms often make it difficult for clients to attend college or hold down a job. Therefore, success in these areas may only be possible once they have sufficient stability and have completed trauma-focused treatment.

Before moving on to describing our group-based approach to phase one, we will outline core treatment principles that guide our clinical approach.

## Core principles underlying our treatment model

### Human rights commitment

Most of our clients have been victims of gross human rights violations and abuse, in particular torture. It is widely recognised that one aim of torture is the control and repression of an individual's basic human rights (Fabri, 2001; Gorman, 2001). Torture and other human rights atrocities are usually carried out within a culture of impunity and the perpetrators seldom acknowledge that this has happened.

In our view, therapists working with this client group must demonstrate a clear commitment to human rights. A neutral therapeutic stance may be seen as colluding with silence, impacting negatively on the development of trust and safety in the therapeutic relationship.

Clients need to feel that clinicians can bear witness to and validate their experiences. The process of talking through these events and having the truth recognised by the therapist can help the individual reclaim their dignity and legitimise their suffering. Where appropriate, clinicians and clients may work with human rights organisations such as Redress and Amnesty International, to help gain wider acknowledgement of these injustices.

### Systemic approach

RAS clients frequently present with multiple needs, necessitating an integrated and whole systems approach. This involves working closely with other relevant organisations and services and may involve for example, writing reports in support of housing and asylum applications. Clinicians also need to recognise and manage the systemic impact of the refugee experience on the family and the wider social network.

### Flexibility

Clinicians need to flexibly adapt treatment according to the changing needs of the client. When external stressors related to issues such as asylum status, health difficulties and family reunification impact on treatment, clinicians may have to stop trauma-focused treatment and return to the task of establishing a sense of trust and safety before any memory processing can proceed. Breaks in treatment or intermittent support may be required whilst other issues are addressed.

### Collaborative work

Another core feature of this work is the principle of mutual collaboration and co-operation. It is generally recognised and corroborated by empirical research that the therapeutic relationship is correlated with successful treatment (Gilbert & Leahy, 2009). Trauma theorists and researchers have also emphasised that the therapeutic relationship is a critical component to successful trauma work (Allen, 2001; Cloitre et al., 2002).

This is especially important for RAS clients who have come from situations in which people in positions of authority have abused their power. Treatment goals can only be achieved within the context of a trusting therapeutic relationship. As far as possible, therapists need to offer choice and allow the client to play an active role in determining treatment goals and process in order to help facilitate a sense of empowerment. Clinicians must demonstrate respect for cultural, religious, and gender diversity, as well as for the clients’ strength and resilience.

### Organisational containment

It is well documented that trauma therapists are at risk of vicarious traumatisation (Figley, 2002; Pearlman & Caringi, 2009). This is especially true when clinicians are working with RAS clients where there is frequent exposure to stories of extreme and deliberate interpersonal violence. We find a supportive team, regular supervision, manageable caseloads and space to talk about the impact of the work all crucial to feeling contained, supported, and validated in this work.

## Phase one: symptom management and stabilisaton group

Symptom management is offered in a time-limited group programme, based on a cognitive-behavioural model. This involves weekly sessions of 2 hours, over 5–8 weeks. Groups consist of up to 10 people of mixed gender, with at least 3 people of each gender. Brief pre-group interviews are conducted in order to familiarise clients with group facilitators, to assess participants’ suitability for group work and to plan for any concerns, which might arise.

Group sessions are preceded by a 30-min drop-in session, to address external problems, such as financial or legal worries, freeing clients to focus on the content of group sessions.

Groups are usually conducted with two group facilitators and up to two trained interpreters. We aim to have at least two group members speaking the same language, so that non-English speakers are not isolated. We use the same interpreter throughout, and adhere to guidelines for working with interpreters (e.g., Tribe & Raval, 2002). We include interpreters in pre-session planning meetings and post-group reviews.

After an initial introductory session, we offer modules including: the management of: flashbacks and dissociation; nightmares and sleep difficulties; hyper-arousal and associated anxiety symptoms; distress and difficult emotions such as anger, and shame; and adapting to life in the United Kingdom.

Clients are initially invited to name problems they experience. These are normalised and organised into a basic problem formulation. The focus then moves to building on skills for managing particular symptoms. These are practiced in each session and then revised and rehearsed in subsequent sessions to increase uptake in clients’ daily lives.

We have observed that due to memory and attention problems, many clients have difficulty recalling much of the information provided. CD recordings and written summaries of sessions can help, as can repeated skills practice.

In our clinical experience, we find that clients seem to benefit most from skills training in managing symptoms of hyper-arousal, anxiety, re-experiencing, and dissociation.

At least one relaxation skill is practiced in each session, and clients are encouraged to practice daily when in a calm state. This includes progressive muscular relaxation, mindful and controlled breathing, and imagery-based “safe place” exercises. We also encourage physical activity. We attempt to match the type of activity to client needs. For clients who experience anxiety in a relaxed state, the toning and strengthening which results from physical activity can have a calming effect (Rothschild, 2000). Clients whose hyper-arousal is triggered by cardiovascular activity are encouraged to try less intense activities such as yoga.

The dissociation management techniques, specifically sensory and cognitive grounding techniques, outlined by Kennerley (1996) and Rothschild (2000) are also extremely beneficial. In our experience, clients will be aware of some triggers for dissociation, but may have difficulty predicting, interrupting or reducing the impact of a dissociative episode. We ask clients to identify known triggers and warning signs that they may be about to dissociate. For example, some clients report physical sensations (such as dizziness) or the presence of a strong emotion (such as fear). We then ask what they already do to ground themselves, and discuss suggestions for adding to these skills. We use resources that facilitate sensory grounding, such as scented oils, smelling salts, soft stress balls, and postcards of London/recent photographs. We identify cognitive updates (e.g., “I am safe,” “I am in London,” “It is 2013”), which are written on a reference card for use as a grounding phrase to help with halting or recovering from dissociative episodes or nightmares (Kennerley, 1996; Rothschild, 2000). It is often necessary to experiment with different techniques in order to find the one that is most helpful. In our experience, a strong smell, as long as it has no negative associations, is most effective.

We will now return to the core domains of complex PTSD and illustrate how difficulties within these areas are addressed in our groups. These will be illustrated with clinical vignettes.^2^

### Difficulties with emotional regulation

Clients with mild to moderate difficulties with emotional regulation are offered symptom management with a focus on distress tolerance techniques (Linehan, 1993) and compassionate mind work (Gilbert, 2005).

Modules of this nature are included in our symptom management groups depending on client need. In these modules, clients are first helped to identify triggers for their overwhelmingly distressing emotions and to understand how these emotions activate their threat systems. There is an emphasis on acceptance rather than blocking of these emotions. Clients are then taught strategies for self-soothing which can include compassionate mind techniques or more concrete, sensory, self-soothing strategies.

#### Case example

Farah, a survivor of childhood abuse, experienced high levels of shame-based distress when reminded of childhood traumas. Triggers for these memories included seeing families and news items related to childhood abuse. In the group, she struggled to develop an image of a “perfect nurturer” (Lee, 2005) or of a safe place (Rothschild, 2000). We find this to be a common problem for clients abused by a trusted person, as the image seems to become contaminated by past abuse. Instead, she was supported to utilise a more concrete approach and to create a “soothing bag” which she filled with valued items, such as a loving letter from her daughter, smooth, semi-precious stones, her favourite perfume, a CD of classical music and a silky shawl which she found comforting (Lee, 2005). With practice she got better at noticing triggers and intervening to self-soothe. Where possible she wrapped herself in the shawl, sprayed her perfume, put on her music, and held the other objects, focussing on the textures, colours, and smells. The immediate, sensory nature of these objects was extremely helpful and she was then able to start using other strategies such as breathing to manage her distress. Adaptations to these strategies worked effectively, such as downloading soothing music onto her phone and keeping the stones and perfume bottle in her bag to be used when out. While Farah benefited from learning these strategies in a group setting, we have found that clients with severe levels of emotional dysregulation, shame, and self-harm, may require individual treatment.

### Disturbances in relational capacities

For some clients, the severity of early traumatic experiences and/or prolonged interpersonal trauma has so seriously disrupted their relational capacities that they may struggle to tolerate being in a group. When this becomes apparent, having two clinicians running the group allows for one to leave the room to support the client in managing their distress before returning to the group. If clients are unable to return to the group, individual symptom management is offered.

#### Case example

Emal, a young Afghan refugee, described a history of frightening and embarrassing panic attacks, and as a result, he was self-isolating and had stopped attending college. During assessment, he told the clinician that he felt unable to trust other people and feared losing control and looking foolish in a group. Two initial individual sessions were offered during which he used thought records to identify triggering situations and beliefs and generated strategies to manage panic symptoms, such as unobtrusive breathing exercises. At his pre-group interview it was agreed that, if required, he could leave the room to do breathing exercises in the bathroom and could indicate if he required support. Once in the group, Emal discovered that others were also experiencing panic and he was able to share his experience of managing his attacks with other group members. His experience of being accepted and affirmed by others in the group was empowering and went some way towards rebuilding his capacity to relate to and trust others.

### Alterations in attention and consciousness

People who have severe difficulties with attention and consciousness struggle to focus on the group content or to benefit from the social elements of a group. In our experience, individual symptom management is necessary and can usefully focus on repeated practice of grounding techniques, with the therapist acting as a close observer of any patterns of dissociation apparent in the session. These sessions can also usefully involve other witnesses to the symptom patterns.

#### Case examples

Anna, a survivor of prolonged civil war, political imprisonment and torture in her home country experienced repeated dissociative episodes throughout the day during which she appeared to suddenly fall asleep. Full neurological investigations at a sleep disorders clinic indicated no underlying organic cause. Anna had difficulty providing a full picture of her daily functioning and of how the dissociation was affecting her. Her partner kept a diary documenting what he noticed and suspected triggers for her dissociative episodes. This allowed the therapist to develop an initial formulation with Anna and her partner that certain difficult emotions, particularly fear, might be triggering these dissociative attacks. They were then able to develop a plan to implement grounding techniques. This included repeated in-session practice of grounding techniques so that the skill became “over-rehearsed.” Anna then practiced these at home with the aid of her partner. This was followed, with Anna repeatedly exposing herself to low levels of fear, by grounding.

Mohammad, a torture-survivor, had learned to ground himself out of flashbacks by burning himself with cigarettes. He also burned himself with a lighter, and at times had pulled out his teeth. He and his therapist traced this behaviour back to his time as a prisoner when he had been forced to harm himself in this way. Mohammed observed that this behaviour only happened when he was in a dissociated state. Mohammad's wife was concerned about the impact of his behaviour on their son, prompting him to find alternative grounding strategies. Mohammed found smelling salts effective. His wife was invited to help with this when she observed him dissociating. He also used stress balls at times that he was sitting quietly, as this was a high-risk time for him to dissociate. Due to the pattern of re-enactment of some of his self-harming behaviour, he also used cognitive updates on note cards to remind himself that he is safe now, that he does not have to hurt himself now, and that he can look after his body.

### Somatic distress and disorganisation

Many clients benefit from psychological formulation of somatic problems, and a graduated approach to addressing the difficulties, which have the greatest impact on their daily lives. For example, people who have extreme chronic sleep problems may benefit from engaging in safe and relaxation-based activities. The initial aim may be to increase understanding and reduce emotional distress related to the sleep pattern, and identify activities that offer some relief and comfort during the night. Some of our clients report patterns of behaviour at night that may be harmful or dangerous (e.g., spending the night outside).

#### Case example

When Phuong, a trafficking victim, felt particularly lonely her sleep deteriorated. To tire herself out, she would start cleaning her flat. However, this triggered memories of traumatic childhood events. She would begin to feel frightened that she could not clean it properly, and would then clean all night in an unsuccessful attempt to reduce her fear. The initial intervention involved identifying alternative activities, so that whilst awake, Phuong could engage in a pleasant activity that would distract her, reducing her likelihood of initiating a cleaning task. Phuong began to look at photos of loved ones, and followed this with watching a nature documentary.

## Reflections and challenges

As discussed previously, establishing the necessary sense of safety in RAS clients is complicated by problems associated with asylum status; with obtaining housing and financial support; with separation from and in some cases the disappearance of family members; as well as loss of culture, language and broader support networks which result in social isolation and further loss of capacity for self-efficacy (Carswell et al., 2011; Grey & Young, 2007; Silove, 1999).

Stabilisation work with clients struggling with these issues necessarily involves supporting them to obtain the appropriate social and legal support to feel practically safe and secure in their external environments, before or at best whilst, supporting them to strengthen their internal psychological resources and strengths. We find that signposting and support in these areas can make a substantial impact on building such clients’ capacities to trust the integrity of our service and of individual clinicians, thus making an important contribution to achieving the goals of phase one work.

The current context of social welfare and immigration reforms within the United Kingdom and a lack of understanding and at times, hostility towards RAS clients, places increased pressure, and demands on clinicians. Creating mutually supportive relationships with third sector organisations is paramount in order to address these shortfalls and can help to ameliorate clinicians’ sense that they have sole responsibility for trying to address failures within the broader social justice system.

The decision as to when to move on to the second phase of treatment must be agreed collaboratively. This often involves a delicate balancing act in which the clinician needs to assess readiness whilst respecting the client's choice and not colluding with their avoidance.

Whilst the group experience can be very powerful in normalising PTSD reactions for clients and reducing social isolation, some clients find the group setting overwhelming, necessitating individual stabilisation work. It is also difficult to focus on specific individual needs of group members within a larger group, due to time constraints and issues of trust and safety. Therefore, some clients may need supplementary individual sessions to address issues, such as intense shame and guilt.

Another challenge relates to the management of behaviours that may be frightening to other group members such as severe dissociation and aggression. In our experience, it is sometimes necessary to remove a member from the group altogether in the interests of his or her sense of safety and containment and that of other members. This needs to be done with sensitivity so as not to communicate the idea that the therapist is unable to tolerate intense distress. Screening and pre-group interviews are helpful in identifying potential difficulties and ensuring group cohesion.

Throughout the group process, it is emphasised that participants should not disclose trauma histories in order to keep the group a safe space. This can be challenging for some clients who have a strong urge to unburden.

A barrier to group treatment is the mistrust commonly experienced by RAS clients towards others from their communities, including interpreters. Careful thought to group composition and choice of interpreter can ameliorate this.

Finally, there is increasing pressure on services such as ours to provide shorter term treatment. This can present difficulties when working with RAS clients who may require lengthy periods of stabilisation.

## Conclusion

RAS clients may present with a range of psychosocial needs and complex PTSD, requiring an integrated and holistic treatment approach. In our experience, a phased intervention is an effective model of treatment for working with this client group in the clinical setting. Whilst there is growing evidence for phase two (exposure-based) treatment interventions for complex PTSD, there is a paucity of literature regarding the effectiveness or contribution of phase one interventions (symptom management and stabilisation) for RAS clients with complex PTSD as described in this article.

There is a need for research to evaluate phased treatment models with this client group. Given the particular experiences and insecurities of this client group, we consider it particularly important to identify the core components of phase one group treatment contributing to clinical improvement.

## Footnotes

1 National Health Service.

## Footnotes

2 Case examples are anonymised composite cases.

## References

[CIT0001] Allen J. G (2001). Traumatic relationships and serious mental disorders.

[CIT0002] Bisson J. I, Ehlers A, Matthews R, Pilling S, Richards D, Turner S (2007). Psychological treatments for chronic post-traumatic stress disorder: Systematic review and meta-analysis. British Journal of Psychiatry.

[CIT0003] Carswell K, Blackburn P, Barker C (2011). The relationship between post-migration problems and the psychological wellbeing of refugees and asylum seekers. International Journal of Social Psychiatry.

[CIT0004] Cloitre M, Courtois C. A, Carapezza R, Stolbach B. C, Green B. L (2011). Treatment of complex PTSD: Results of the ISTSS expert clinician survey on best practices. Journal of Traumatic Stress.

[CIT0005] Cloitre M, Courtois C. A, Ford J. D, Green B. L, Alexander P, Briere J (2012). The ISTSS expert consensus treatment guidelines for complex PTSD in adults. http://www.istss.org.

[CIT0006] Cloitre M, Koenen K. C, Cohen L. R, Han H (2002). Skills training in affective and interpersonal regulation followed by exposure: A phased-based treatment for PTSD related to childhood abuse. Journal of Consulting and Clinical Psychology.

[CIT0007] Cloitre M, Stovall-McClough K. C, Nooner K, Zorbas P, Cherry S, Jackson C. L (2010). Treatment for PTSD related to childhood abuse: A randomized controlled trial. American Journal of Psychiatry.

[CIT0008] Crumlish N, O'Rourke K (2010). A systematic review of treatments for post-traumatic stress disorder among refugees and asylum seekers. Journal of Nervous and Mental Disease.

[CIT0009] Ehlers A, Clark D. M (2000). A cognitive model of post-traumatic stress disorder. Behaviour. Research and Therapy.

[CIT0010] Fabri M (2001). Reconstructing safety: Adjustments to the therapeutic frame in the treatment of survivors of political torture. Professional Psychology: Research and Practice.

[CIT0011] Figley C. R (2002). Treating compassion fatigue.

[CIT0012] Gilbert P (2005). Compassion: Conceptualisations, research and use in psychotherapy.

[CIT0013] Gilbert P, Leahy R. L (2009). The therapeutic relationship in the cognitive behavioral psychotherapies.

[CIT0014] Gorman W (2001). Refugee survivors of torture: Trauma and treatment. Professional Psychology: Research and Practice.

[CIT0015] Grey N, Young K (2007). Cognitive behaviour therapy with refugees and asylum seekers experiencing traumatic stress symptoms. Behavioural and Cognitive Psychotherapy.

[CIT0016] Herman J. L (1992). Trauma and recovery. From domestic abuse to political terror.

[CIT0017] Kennerley H (1996). Cognitive therapy of dissociative symptoms. British Journal of Clinical Psychology.

[CIT0018] Kruse J, Joksimovic L, Cavka M, Woller W, Schmitz N (2009). Effects of trauma-focused psychotherapy upon war refugees. Journal of Traumatic Stress.

[CIT0019] Lee D. A, Gilbert P (2005). The perfect nurturer: A model to develop a compassionate mind within the context of cognitive therapy. Compassion: Conceptualisations, research and use in psychotherapy.

[CIT0020] Linehan M. M (1993). Cognitive behavioural treatment of borderline personality disorder.

[CIT0021] NICE (National Institute for Clinical Excellence) (2005). Post-traumatic stress disorder (PTSD): The management of PTSD in adults and children in primary and secondary care.

[CIT0022] Nickerson A, Bryant R. A, Silove D, Steel Z (2011). A critical review of psychological treatments of posttraumatic stress disorder in refugees. Clinical Psychology Review.

[CIT0023] Pearlman L. A, Caringi J, Courtois C. A, Ford J. D (2009). Living and working self-reflectively to address vicarious trauma. Treating complex traumatic stress disorders: An evidence-based guide.

[CIT0024] Rothschild B (2000). The body remembers. The psychophysiology of trauma and trauma treatment.

[CIT0025] Schauer M, Neuner F, Elbert T (2005). Narrative exposure therapy—A short term intervention for traumatic stress disorders after war, terror or torture.

[CIT0026] Shapiro F (1995). Eye movement desensitisation and reprocessing: Basic principles, protocols and procedures.

[CIT0027] Silove D (1999). The psychosocial effects of torture, mass human rights violations, and refugee trauma: Towards an integrated conceptual framework. Journal of Nervous and Mental Disease.

[CIT0028] Tribe R, Raval H (2002). Working with interpreters in mental health.

[CIT0029] Van der Kolk B. A, Roth S, Pelcovitz D, Sunday S, Spinazzola J (2005). Disorders of extreme stress: The empirical foundation of a complex adaptation to trauma. Journal of Traumatic Stress.

